# Synchronous Rhythmic Interaction Enhances Children’s Perceived Similarity and Closeness towards Each Other

**DOI:** 10.1371/journal.pone.0120878

**Published:** 2015-04-08

**Authors:** Tal-Chen Rabinowitch, Ariel Knafo-Noam

**Affiliations:** 1 Department of Psychology, Hebrew University of Jerusalem, Jerusalem, Israel; 2 Institute for Learning and Brain Sciences, University of Washington, Seattle, Washington, United States of America; Max Planck Institute for Human Cognitive and Brain Sciences, GERMANY

## Abstract

Inter-personal synchronization is important for performing many cooperative tasks. Notably, synchrony has also been shown to have considerable positive social influences, possibly mediated by synchrony-induced changes in social attitude such as an increased sense of similarity and affiliation between interacting individuals. This effect has been demonstrated in adults, but it is unknown whether synchrony might have a similar impact on the social attitudes of children. We thus set to directly examine the influence of synchronous rhythmic interaction on perceived similarity and closeness in pairs of 8–9 year old children. We found that children who had participated in a synchronous interaction regarded their interacting partner as more similar and closer to themselves than children who had not interacted at all or who had taken part in an asynchronous interaction. These findings reveal that synchronous interaction can positively alter social attitudes between interacting children, suggesting a potential mechanism by which synchrony may enhance positive social interaction through attitudinal shift.

## Introduction

The propensity to cooperate is a hallmark of human civilization, essential for productivity and survival. One exceptionally prominent basis for inter-personal coordination is synchrony [1], the temporal aligning of action between two or more interacting individuals. At its most fundamental level, synchrony is simply a physical prerequisite for the successful execution of collaborative timing-dependent activities, such as rowing or playing music together. However, being in synchrony appears to have an impact that extends beyond the synchronous interaction itself. It can lead to improved performance in a subsequent cooperative activity [2], and it has a positive impact on interactants’ overall prosocial behavior, exhibited for example, as an increased inclination to offer help to an interacting partner [3] or to cooperate with her [4].

Several studies have demonstrated that individuals, even passively receiving sensory stimulation in synchrony with another person, tend to experience a form of self-other merging with that other person. For example, participants viewing a morphed image of their own face and another’s [5], or a stranger’s face [6–7], tended to recognize the other's face as their own or to experience resemblance and closeness to the unfamiliar face after a period of synchronous stroking of their face and the face that they were watching. Such an enhanced sense of similarity and affiliation may also emerge during active synchronous interaction such as between two participants rocking synchronously on rocking chairs [2], or between a participant and an experimenter tapping together in synchrony [8–9].

These examples demonstrate the potential of synchrony, to enhance a sense of similarity and affiliation among interacting individuals [1, 10], raising the possibility that similarity may serve as a mediating factor between synchrony and positive social interaction [9]. Several studies seem to support this premise. For example, similar interactants expressed more liking towards one another [11], were more likely to engage in a smooth, conflict-free interaction [12], had a better relationship quality [13] and tended to communicate more effectively with each other [14]. Perceived similarity, and specifically that of personal values and traits has been shown also to act as an antecedent to rapport [15].

All work done so far on the effects of synchrony on perceived similarity and closeness has been performed on adults. However, synchronous interaction occurs naturally already at early infancy between caregiver and infant in the form of temporal coordination of nonverbal behaviors such as gaze, affect, vocalizations, body movements, and several arousal indicators [16–18]. Moreover, engagement in synchronous interaction has been shown to influence the behavior of children and even very young infants. Thus, newborns were shown to spend more time looking at an image of a face being stroked in synchrony rather than asynchrony with their own face [19]. 14 month-old infants were shown to increase their helpfulness behavior towards an experimenter with whom they experienced synchronous interaction [20]. More generally, children engaging in joint musical activity, a form of interaction rich in synchrony, displayed increased pro-social behavior [21] and an enhanced capacity for empathy [22]. We thus wished to determine whether attitudinal shifts such as an increased sense of similarity and closeness that have been repeatedly observed in adults in conjunction with interpersonal synchrony might occur also in children. The existence of such an effect in children would suggest a fundamental role for attitudinal change in shaping the behavioral outcomes of interpersonal synchrony.

To address this question we examined synchronous rhythmic interaction in an active setting comprising two interacting 8-year-old participants. We found that children participating in a brief synchronous rhythmic interaction perceive themselves to be more similar and feel closer to their interacting partner than children who had engaged in an asynchronous interaction or children who had not engaged in any interaction at all.

## Methods

### Ethics statement

This research was carried out under the approval of the Hebrew University of Jerusalem Social Sciences Review Board. Written parental consent and individual assent was gained for all children who volunteered to participate.

### Participants

Participants were 74 dyads of same-sex, previously unacquainted 8 year-olds (46 girl pairs; 28 boy pairs; M_age_ = 8.6 years, SD = 3.6 months). All participants were part of a larger study pertaining to the Longitudinal Israeli Study of Twins (LIST) [23], and were thus one of a twin pair (for the present study, twins were separated so that each dyad was composed of non-sibling participants; the children in the dyad were completely unknown to each other prior to the study). The fact that participants were twins was of no relevance to the study reported here. A total of 52 children (22 monozygotic and 30 dizygotic twins) were included in a synchrony interaction, 56 children (22 monozygotic and 34 dizygotic twins) participated in an asynchronous interaction, and 40 children (20 monozygotic and 20 dizygotic twins) did not engage in any interaction. The latter group was added to the design *post hoc* in order to establish a baseline. This *post hoc* study was performed 12–16 months after the synchrony and asynchrony conditions were tested. Participants in all conditions were of similar age and background, taken from the same cohort of subjects. Other than the interaction, all experimental procedures were identical across groups and were carried out by the same experimenters.

The choice of age group was made according to the following considerations. On the one hand the task in our study (see below) consisted of tapping according to the pace of individually presented isochronous visual stimuli, an ability [24] that emerges by about 6 years of age and further improves as the child develops [25]. On the other hand, our study participants were required to tap together with a partner and to thus unknowingly engage in either synchronous or asynchronous interaction, similarly to [8]. Since the ability to synchronize with a rhythmic sequence continues to develop through childhood and only reaches adult levels of performance by around the age of 10 [25], we reasoned that in order to experience the synchronous interaction and to successfully perform the task, participants should be closer in age to 10. We thus set the age group to range between 8 and 9 years of age.

### Procedure

Participants were invited to the lab for a series of experiments, one of which is reported here. The children were seated in a quiet room. The experimenter introduced herself and asked the children to introduce themselves by names only. The experimenter explained to the children who had been pre-assigned to synchronous or asynchronous interaction that she is interested in learning about how children play together, and that she is especially interested in rhythm. Then, the experimenter showed the children how the tapping task should be done and each child practiced the task separately. After both children felt comfortable with the task, they performed it, in two blocks of 1.5 minutes each. Following the rhythmic interaction, each child individually completed a similarity questionnaire and a closeness measure. The children not participating in any interaction were directly instructed to complete the questionnaire.

### Experimental Tasks and Materials

#### The tapping task

**Fig 1:**
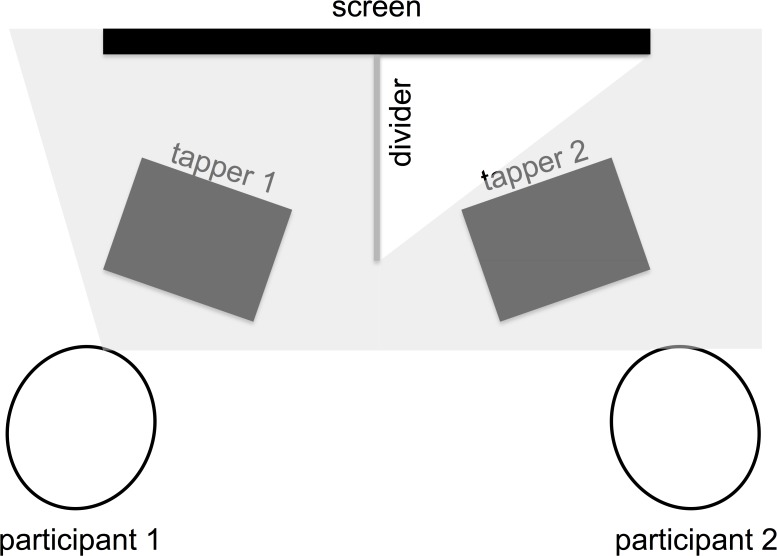
Experimental setup. Two children were seated side by side in front of a computer screen and two tappers. A divider was placed on the screen so that each child could only see his or her half of the screen. The other child and his or her tapper were visible to each participant. The shaded area represents the field of view of one of the participants.

The tapping task was designed to encourage participants to implicitly and unintentionally engage in one of two interaction types: synchrony or asynchrony. Each child dyad was randomly assigned to either one of these conditions (or to no tapping at all). The tapping was performed on an electrical tapper, a hand-played electronic percussion controller with 4 individual pads (Alesis PercPad). Children sat next to each other, each facing the electric tapper and a shared video screen divided in the middle, so that each child only saw their half of the screen, but could see the other child’s tapping (Fig. 1). In both types of interaction the children tapped together according to a bouncing ball appearing on each child’s half screen. The ball jumped up and down in an isochronous manner (constant interval between each bounce) and the children were asked to tap each time the ball reached the floor. To make this moment more distinguishable, the floor also turned red at the time of each expected tap. In addition, the ball moved according to a cosine function to give it a realistic appearance [26]. No auditory stimulus accompanied the bouncing ball, so that the only auditory feedback the children received both in the synchronized and asynchronized conditions was their own and their partner's tapping. In this way the children’s sensorimotor experience had the same auditory delays, and consisted of the same basic units of IOI. This was done so that participants’ aural attention should be directed as much as possible to the outcome of the rhythmic interaction. Children performed two blocks of 1.5 minutes long rhythmic interactions with a brief interval between them for resting the hands. In each of the blocks, the children tapped at either a fast frequency, of 600ms Inter-Onset Interval (IOI) between taps, or a slower rate of 800ms IOI between taps. These frequencies appeared to be comfortable to follow according to an earlier pilot whereby we presented children with different IOIs, asked them to tap along with auditory, as well as visual stimuli, and then monitored their success in tapping with the stimuli. We also asked participants how easy or difficult it was for them to follow the beat and tap. Most of the children felt comfortable around the 700ms IOI range. In the synchrony condition, both children tapped in phase at the same frequency within a block (i.e. Block 1: Child 1, 800 IOI; Child 2, 800 IOI; Phase 0 degrees. Block 2: Child 1, 600 IOI; Child 2, 600 IOI; Phase 0 degrees). In the asynchrony condition, each child tapped at a different frequency in each block, so the IOIs always had an 800/600 (1.33) ratio between them. In addition, tapping was performed at a 180 degrees phase difference (one of the participants’ tapping times were shifted by half of his or her IOI), further emphasizing the asynchrony (i.e. Block 1: Child 1, 800 IOI; Child 2, 600 IOI; Phase 180 degrees. Block 2: Child 1, 600 IOI; Child 2, 800 IOI; Phase 180 degrees). The children found the tapping task simple and easy to perform after a short practice, whereby each child individually tapped on the electrical tapper with the bouncing ball. For most children, the practice lasted about a minute or so. Children who needed more time to practice were given the chance to practice some more. When both children were ready, the actual rhythmic interaction began. The experimenter explained to the children that now both of them are going to tap at the same time for 1.5 minutes twice – “Now we will do the same as we just practiced, but you will play at the same time”. The rhythmic interaction was recorded on a Lenovo ThinkPad Edge 520 laptop, using Reaper Digitial Audio Workstation (Cockos Incorporated).

#### Measure for synchrony

In order to confirm that the synchrony and asynchrony conditions indeed resulted in synchronous and asynchronous interactions, respectively (manipulation check), we analyzed the recorded tapping sequences using the SPIKE-dist measure, originally developed for assessing the degree of synchrony between two or more trains of neuronal action potentials [27]. Given two tapping sequences, the SPIKE-dist measure averages for each time instant the absolute differences between the previous tap times and the following tap times and normalizes this average by the mean length of the inter-tap intervals. Its values range from 0, perfect synchrony, to 1, perfect asynchrony. This measure is especially suitable for comparing two dynamic and noisy tapping streams (rather than, for instance, a fixed metronome and a participant follower). In addition, the measure is parameter free and timescale independent. This measure has already been used successfully in several studies (e.g. [28–29]). In order to compute SPIKE-dist, the MIDI files created during the tapping sessions were imported to Matlab (Mathworks, Natick, Mass) and the tap times starting from 10sec after the beginning of the interaction (warm up time) till the end of the interaction were extracted separately from the two participant channels. Many participants had occasionally accidently tapped on more than one pad per intended tap. Indeed, during the analysis, when we examined the distribution of the tapping intervals (S1 Fig.) we found in addition to the expected peak at the designated IOI, an additional peak close to 0sec, which was well separated from the main peak. We thus set 100ms, safely distant from both peaks, as a cutoff for non-single taps, and removed any tap events that occurred at a shorter time interval after a preceding tap. We used a Matlab implementation of the SPIKE-dist measure (http://wwwold.fi.isc.cnr.it/users/thomas.kreuz/sourcecode.html), with a temporal resolution of 1ms, to analyze our data.

#### Similarity

In order to evaluate participants’ perceived similarity to their dyad partner we used a self-report questionnaire. The perceived similarity questionnaire consisted of 6 Likert-type scale questions, ranging from 1 (not similar at all) to 4 (extremely similar). The questions targeted general similarity (Q.1–2); similarity in appearance (Q.3); similarity in character (Q.4); similarity in hobbies (Q.5); and similarity in music styles (Q.6). Examples of questions included: “Does he/she remind you of yourself in any way”? Do you think he/she is similar to you in character”? “Do you think he/she likes the same musical styles that you do”? The perceived similarity score was the average rating of all questions (Cronbach’s α = 0.73). In order to validate this measure we asked a separate group of monozygotic (MZ; identical) and dizygotic (DZ; non-identical) twins to fill in the questionnaire regarding their sibling twin, expecting the MZ twins, which are highly similar to each other, to obtain higher similarity scores than the DZ twins. MZ twins have previously been shown to report being more similar to each other than DZ twins in behavior as well as in appearance [30–31]. Indeed, MZ twins perceived their twin sibling to be more similar to themselves (M = 3.0, SD = 0.42) than did DZ twins (M = 2.0, SD = 0.44, t(23) = 5.68, p<0.001, d = 2.3).

#### Closeness

To examine how close the tapping partners felt towards each other after tapping we adapted for our study the Inclusion of Other in Self (IOS) scale, originally designed for gauging relationship closeness between adults [32], similarly to [33]. Children were presented with a series of pairs of circles, the first labeled ‘me’ and the second labeled either ‘he’ or ‘she’, depending on the sex of the pair, with an increasing degree of overlap between them. Each child was asked to select the pair of circles that best represents his or her experience of tapping together: “Out of the following options, how did you feel during the joint tapping on the electric pads (please choose the most relevant option)?” The score ranged from 1 (little or no closeness) to 6 (high level of closeness). The rationale was that children who experienced a strong sense of togetherness will select one of the more overlapping pairs of ‘me’/‘he-she’ circles, whereas children experiencing a weaker sense of togetherness will select a more separated pair of circles. When twins not participating in the current study were asked to indicate how they felt one towards the other when tapping together, the difference between MZ (M = 5.2, SD = 0.85) and DZ (M = 4.3, SD = 1.62) siblings in IOS closeness scores was not statistically significant (t(21) = 1.56, p = 0.108, d = 0.7).

## Results

**Fig 2:**
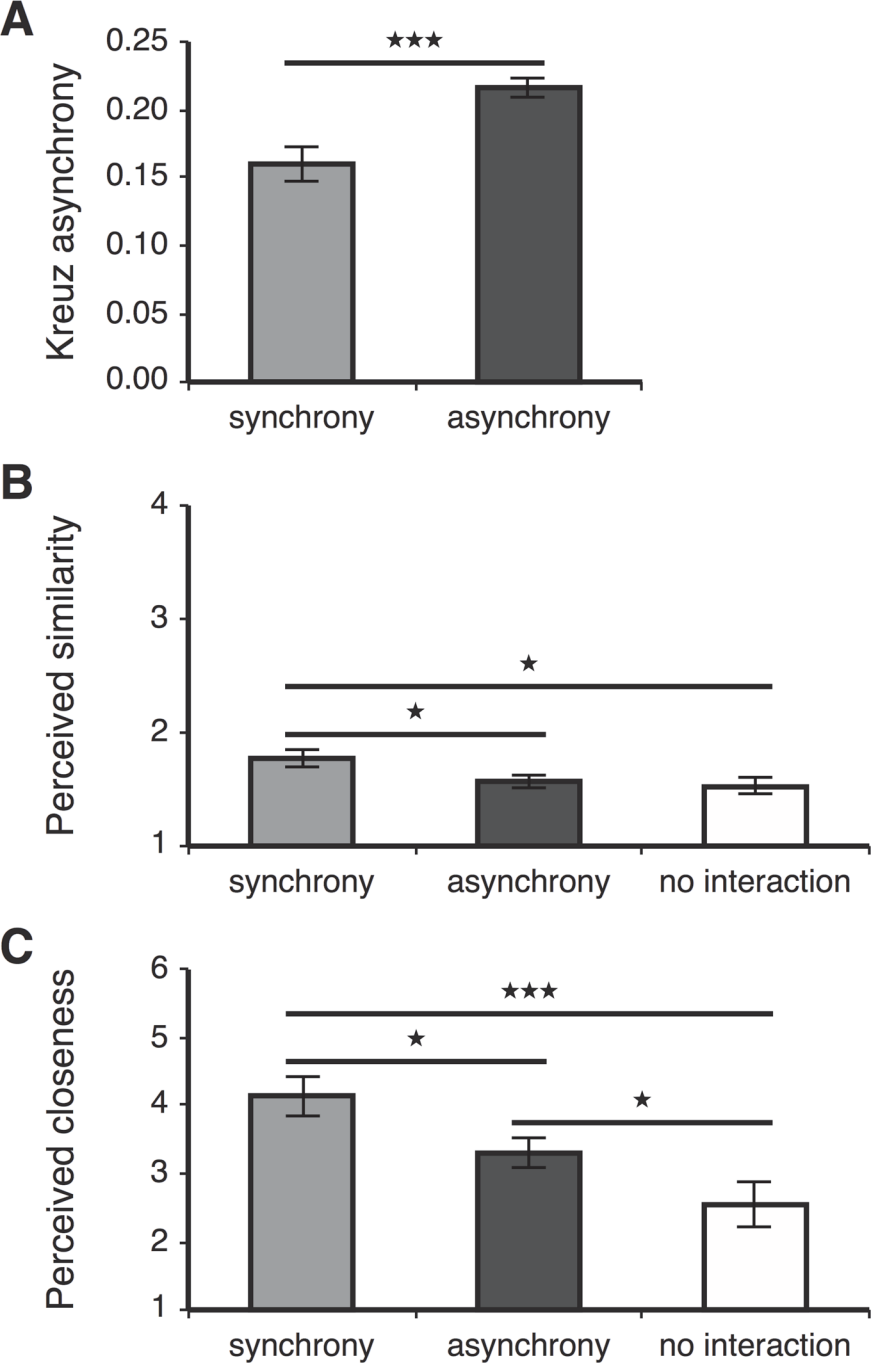
Mean Kreuz asynchrony score. (A), perceived similarity score (B) and perceived closeness score (C) for synchronous and asynchronous interactions, or no interaction. Error bars are the standard errors of the mean. *p<0.05, ***p<0.001 relative to synchrony condition in unpaired two-tailed t-tests not corrected for multiple comparisons.

We sought to determine whether there was a difference in perceived similarity or closeness between child participants who had engaged in a synchronous versus asynchronous rhythmic interaction or had not engaged in any interaction at all. However, we first wished to confirm that interacting participants had indeed experienced the type of interaction designated for them. To this end we applied the Kreuz SPIKE-dist measure to the recorded tapping sequences (see Methods), and found that indeed, the intended asynchronous interactions received a significantly higher asynchrony score (M = 0.22, SD = 0.04) than the synchronous interactions (Fig. 2A; M = 0.15, SD = 0.06, t(51) = 4.26, p<0.001, d = 1.2).

We thus proceeded to analyze participants’ perceived similarity to their dyad partner. First, however, we wished to determine whether dyad members could be considered statistically independent. If that is the case then each individual participant can be considered as the unit for analysis, and the sample size is the total number of participants. To this end we computed the intra-class correlations [34] of all three groups (synchrony, asynchrony and no interaction) and performed an F-test to compare the within dyad and between dyad variances. For all groups the result was not statistically significant (F(24,25) = 1.21, p = 0.321, F(28,27) = 1.1, p = 0.401, F(28,27) = 1.05, p = 0.452, respectively), confirming the statistical independence of participants. This allowed us to compare between the individual synchrony, asynchrony and no interaction similarity scores (Fig. 2B). One-way ANOVA revealed that perceived similarity differed significantly across the three conditions (F(2,143) = 3.638, p = 0.029). Post hoc t-tests indicated that perceived similarity scores following synchrony (M = 1.77; SD = 0.53) were significantly higher than following asynchrony (M = 1.57, SD = 0.43, t(104) = 2.16, p = 0.035, here and thereafter not corrected for multiple comparisons, d = 0.42) or following no interaction at all (M = 1.53, SD = 0.43, t(88) = 2.33, p = 0.022, d = 0.49). Thus, synchronous interaction resulted in a stronger perceived similarity.

To reveal whether synchronous interaction might also induce a sense of closeness between interacting partners, we examined the Inclusion of Other in Self (IOS) scores. Unlike perceived similarity, we found significant intra-class correlations within synchrony and no interaction dyad members (F(24,25) = 3.25, p = 0.002, F(27,28) = 7.53, p<0.001, respectively), but not within asynchrony dyad partners (F(27,28) = 1.05, p = 0.449). This indicated that in the synchrony and no interaction groups, closeness scores between dyad members were correlated. We thus considered as the unit of analysis for all three conditions the mean closeness score of each dyad (Fig. 2C). One-way ANOVA revealed a significant difference across the three conditions (F(2,69) = 8.43, p = 0.001), and post hoc t-tests showed a significantly larger closeness score for synchrony (M = 4.14, SD = 1.45) compared to asynchrony (M = 3.3, SD = 1.12, t(51) = 2.35, p = 0.022, d = 0.65) or no interaction (M = 2.47, SD = 1.48, t(42) = 3.74, p<0.001, d = 1.14). Interestingly, the closeness score was also significantly larger for asynchrony compared to no interaction (t(45) = 2.08, p = 0.040, d = 0.65). Thus, merely tapping together had a positive impact on perceived closeness, which was especially enhanced when the tapping was synchronous.

## Discussion

We have found that 3 minutes of synchronous interaction were sufficient for children to feel similar and close to their interacting partner. The similarity and closeness scores of children engaged in asynchronous interaction or no interaction at all were significantly lower. These findings emphasize that already in children synchrony can influence social attitude, a potential precursor of pro-social behavior.

Although synchrony resulted in an enhanced sense of both similarity and closeness, these two aspects of social attitude were distinguishable, as revealed when comparing the effects to the baseline condition of no interaction. Whereas similarity scores were similar between asynchrony and baseline (Fig. 2B), closeness scores were higher for asynchrony than baseline (Fig. 2C). These results suggest that similarity to self might be more selectively boosted by synchrony than feelings of closeness. The exclusive effect of synchrony on similarity could have resulted from the physical resemblance of the auditory and visual feedback that the children were receiving from both tappers and from their coinciding hand movements. Such physical coordination might extend to social-attitudinal resemblance, making the children assume that whoever is *acting* like me, is probably *like me*. This notion is in accord with the ‘Like Me’ framework, whereby infants being mimicked tend to interpret the others’ psychological states (i.e. perceptions and emotions) as being similar to their own [35]. Closeness was also more pronounced after synchrony compared to asynchrony, possibly for similar reasons. However, the finding that even asynchronous tapping resulted in more closeness than no tapping at all could be due to the mere shared experience of tapping.

How can similarity or closeness lend themselves to more positive social interaction? As already discussed, several studies support the link between similarity and positive social interaction in adults [11–14], possibly also via the enhancement of rapport between interactants who feel similar to each other [15]. In addition, a sense of similarity may elevate one’s motivation and capacity to experience the other person more from the first-person perspective than from the third-person perspective, and in turn, this kind of simulation can potentially be a precursor for empathy [36], possibly culminating in more pro-social behaviors. These findings together with those reported here lend support to the notion that feelings of self-similarity are the root, or at least part of the root for the pro-social behavioral outcomes found after interpersonal synchronization [9]. At the same time, elevated levels of closeness, which is in itself a characteristic of social interaction [37] may be expected to result in more positive, pro-social behaviors as well.

Similarly to previous studies on adults (e.g. [3, 8, 9, 38]) our participants experienced synchronous interaction with one another without any intention or explicit instruction to do so. Nevertheless, the mere experience of acting in synchrony with another was sufficient to alter the sense of similarity and closeness to the other. The naturalistic approach of the study, examining the relationship between synchrony and perceived similarity and closeness in a real-time active interaction between two participants rather than, for example, a participant and an experimenter, increases the applicability of our findings to real-world situations.

In previous work we have shown that children participating in weekly musical group interaction sessions, which focused on particular features of musical interaction, including synchrony, improved their capacity for empathy compared to children from control groups [22]. The present study provides specific evidence for the positive effects that synchronous interaction may have on children. These findings pave the way to further research on synchronous interaction in children and their social influences including their potential role in intervention strategies.

## Supporting Information

S1 Fig Histogram of inter-onset intervals (IOI) for all participants. Red dotted line indicates inclusion threshold. All tapping that occurred less than 100ms after a preceding tap were omitted as they were likely accidental multiple tapping representing a single beat.
(TIFF)
