# Supplementary figures and images for: Synchronous Rhythmic Interaction Enhances Children’s Perceived Similarity and Closeness towards Each Other
Source: PLoS One. 2015 Apr 8;10(4):e0120878. doi: 10.1371/journal.pone.0120878 (PMC4390221; doi:10.1371/journal.pone.0120878)

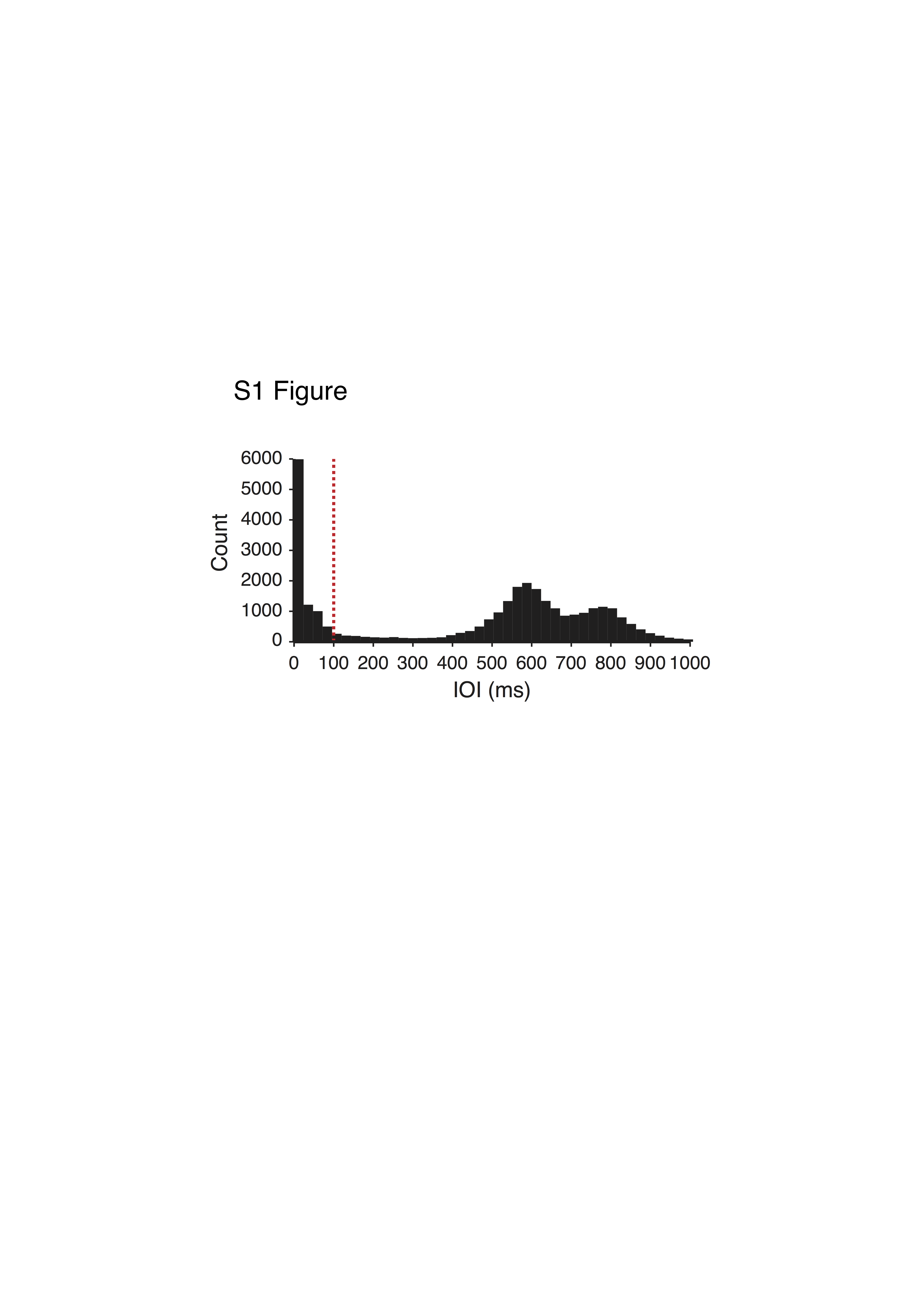

Supplement: S1 Fig — Red dotted line indicates inclusion threshold. All tapping that occurred less than 100ms after a preceding tap were omitted as they were likely accidental multiple tapping representing a single beat. (TIFF) [file pone.0120878.s001.tiff]
